# Pharmacotherapy for sleep during critical illness and beyond

**DOI:** 10.3389/fneur.2026.1814615

**Published:** 2026-04-28

**Authors:** Gerald L. Weinhouse

**Affiliations:** Brigham and Women's Hospital, Harvard Medical School, Boston, MA, United States

**Keywords:** chronotherapy, critical illness, delirium, homeostatic control, hypnotic, pharmacotherapy, circadian

## Abstract

Sleep is a complex process thought to be regulated by both a circadian system closely tied to ambient light and a homeostatic process characterized by increasing pressure to sleep commensurate with increasing duration of wakefulness. Critical illness is often a complex, multi-system physiological insult characterized by high levels of stress hormones and inflammatory biomarkers as well as pain and anxiety which negatively impacts sleep. Sleep loss may potentiate physiological disturbances and is increasingly being linked to poor ICU outcomes. The current gold standard for improving the sleep of ICU patients is to bundle multiple non-pharmacologic interventions; medication should only be prescribed for sleep when those measures failed to produce the desired results. Medication administered to facilitate sleep has had disappointing results in the ICU and may have unintended consequences such as increasing the risk of delirium and falls. In general, pharmacotherapies used to induce sleep (hypnotics) have focused on targeting specific points along the arousal/sleep pathway. Another approach has been to enhance chronotropy both by non-pharmacologic measures as well as by melatonin supplementation or administration of melatonin receptor agonists. There have been some efforts to promote wakefulness as an effort to leverage the homeostatic pressure to sleep at night rather than allowing sleep to occur intermittently throughout the 24 h day as occurs during critical illness. This review will summarize the current data regarding the pharmacologic management of sleep in the ICU and propose one potential approach.

## Introduction

Sleep is a complex process thought to be regulated by both a circadian system closely tied to ambient light and a homeostatic process characterized by increasing pressure to sleep commensurate with increasing duration of wakefulness. Critical illness is often a complex, multi-system physiological insult characterized by high levels of stress hormones and inflammatory biomarkers as well as pain and anxiety which negatively impacts sleep. Challenges to getting sleep while critically ill are further complicated by an ICU environment hostile to sleep and frequent care interventions. Sleep loss may potentiate physiological disturbances and is increasingly being linked to poor ICU outcomes (1).

The current gold standard for improving the sleep of ICU patients is to bundle multiple non-pharmacologic interventions (2); medication prescribed for the primary purpose of helping patients sleep should only be considered when those measures have failed to produce the desired results. Polypharmacy may be harmful to patients in the ICU. On average, critically ill patients are prescribed 8–12 medications (3, 4). It is known that high medication complexity scores are associated with poor clinical outcomes (5–7). Medication specifically administered to facilitate sleep has had disappointing results in the ICU, contributes to polypharmacy, and may have unintended consequences such as increasing the risk of delirium and falls. It is important to be aware that consciousness under infusions of sedative medications is distinct from sleep (8).

In general, pharmacotherapies used to induce sleep (hypnotics) have focused on targeting specific points along the arousal/sleep pathway.

Another approach has been to enhance chronotropy both by non-pharmacologic measures as well as by melatonin supplementation or administration of melatonin receptor agonists. Conversely, there have been some efforts to promote wakefulness as an effort to leverage the homeostatic pressure to sleep at night rather than allowing sleep to occur intermittently throughout the 24 h day as is often the case during critical illness.

This review will summarize the current data regarding the pharmacologic management of sleep in the ICU and propose one potential approach.

### Sleep and delirium pharmacotherapy

The relationship between poor quality sleep during critical illness and ICU delirium is unknown but may be bidirectional. Physiologic functions dependent on sleep may be lost during periods of sleep deprivation as is common in the ICU and may lead to the manifestations of delirium. Sleep disruption is associated with altered CNS electrical activity and organization which may be associated with inefficient clearing of CNS metabolic waste, impaired memory, inattention and poor executive function which are hallmarks of delirium (9). Functional connectivity, the relationship between different regions of the brain measured in synchronized activity rather than anatomic connection, may be altered after sleep loss and has been implicated in poor neuropsychological function (10–12). Both acute sleep loss and delirium have been found to be associated with loss of functional connectivity, especially of the default mode network which is most active during quiet wakefulness and has a critical role in memory and emotional processing (11, 13–15). Conversely, delirium has been associated with dysregulation of melatonin secretion and alterations of the arousal system leading to poor sleep (16).

It is not surprising, in this context, that medications given to improve sleep have been tested for their effect on reducing the incidence of delirium. Systematic reviews of interventions to improve sleep in the ICU, both non-pharmacologic interventions alone and those including sleep bundles which include a pharmacologic option, have found a reduced incidence of delirium (17).

## Physiology of sleep as targets for pharmacotherapy

Both the circadian and homeostatic regulation of sleep is disrupted during critical illness; malalignment of these systems is known to be associated with both acute and long-term effects on cardiovascular, metabolic, and respiratory outcomes (18). A successful approach to improving sleep in the critically ill will almost certainly require interventions that address both. This review will include pharmacotherapies to promote sleep and also those that are chronotropic, i.e., affect the timing of sleep.

### Hypnotics for sleep induction

#### GABA agonists

The ventrolateral preoptic nucleus (VLPO) of the anterior hypothalamus innervates the wake-promoting regions of the brain; gamma aminobutyric acid (GABA) inhibitory activity there leads to sleep onset. GABA is the major inhibitory neurotransmitter in the brain and its receptors have been pharmacologic targets for sleep promotion.

Propofol and, less frequently, benzodiazepines are the primary GABA agonists given for anxiolysis and sedation during critical illness. They both bind to the GABA-A receptor but at sites distinct from each other. Differences in specificity for the GABA receptor subtypes account for the differences in efficacy among the various agents used. There may be overlapping neural networks with sleep; however, available data does not suggest that they improve sleep (19). Ultimately, what is most important is whether the physiologic functions enhanced under natural sleep are also enhanced under sedation.

*Propofol*, in contrast to naturally occurring sleep, is known to produce amnesia and reduce REM in critically ill patients although in healthy adults biomimetic stages N2 and N3 may be increased (20). Importantly, however, slow waves under propofol infusions are distinct from those during NREM sleep (21). Glymphatic flow occurs during propofol infusion similar to that during deep N3 sleep in animal models (22), but it is less clearly quantified in critically ill humans. Clinical studies have failed to demonstrate that critically ill patients sleep better while on infusions of propofol and it is not recommended for that indication alone (2, 19, 20).

*Benzodiazepines* produce amnesia and reduce stages N3 and REM; therefore, they are believed to decrease glymphatic flow (23). Benzodiazepines also alter sleep spindles which are linked with memory consolidation (24). Benzodiazepines are associated with the development of ICU delirium which is associated with an increased risk of mortality so they are avoided when possible (25–27). The benzodiazepine receptor agonists, the so-called “z-drugs” (zolpidem, zopiclone, eszopiclone) used for insomnia in ambulatory adults, may disturb glymphatic clearance due to reduced stage N3 and norepinephrine reduction but have not been studied in the critically ill (28). Like propofol, benzodiazepines have not improved sleep in the critically ill (29).

#### Alpha agonists

*Dexmedetomidine* is a highly selective alpha-2 agonist and is recommended for ICU sedation (30). It is thought to induce a state of consciousness more closely resembling sleep than the other sedative infusions (31–33). N2 and N3 sleep can be observed during infusion with dexmedetomidine in healthy subjects, and sleep spindles are seen (34). Glymphatic flow is enhanced relative to other sedatives possibly owing to its noradrenergic modulation (35–37). But as with all sedative infusions, circadian and sleep stage cycling does not occur, and REM is markedly reduced. Clinical trials have not consistently indicated improvement in sleep quality (38–43). Importantly, however, the incidence of delirium is lower with dexmedetomidine than with other sedative infusions, so it is currently the preferred agent if a sedative infusion is needed, especially for nocturnal sedation (30).

#### Dual-orexin receptor antagonists (DORAs)

*DORAs* inhibit wake-promoting neuropeptides orexin A and orexin B from binding to their receptors. There is very limited data on the effects of DORA's on sleep in the critically ill, but medication such as suvorexant and lemborexant may preserve sleep architecture in healthy adults better than other medications used for sleep in the critically ill (44). They have a favorable safety profile, do not cause amnesia, and several studies have suggested that they can reduce the incidence of ICU delirium (45); however, current data is insufficient as trials have been retrospective or underpowered to be conclusive. One randomized controlled trial of suvorexant vs. placebo for elderly, hospitalized (not exclusively ICU) patients found a reduced incidence of delirium but no demonstrable difference in subjective sleep parameters (46). They are only available for oral administration.

#### Others

*Antidepressants* which inhibit serotonin reuptake are used for sleep but with scant supportive data in the critically ill. They are sedating, which might help patients sleep, but they also have anticholinergic effects with an increased risk of delirium and they have side effects which include orthostatic hypotension and cardiac conduction delays which may be unacceptable in vulnerable critically ill patients.

Trazodone is one of the most commonly used antidepressants in the ICU; however, studies of the use of trazodone for sleep in hospitalized patients are limited, have mixed results, and almost exclusively include non-ICU patients (47, 48) It may be an option for those with insomnia following a critical illness where sleep problems often persist along with mental health challenges, i.e., post-ICU syndrome (PICS). In outpatients, trazodone may increase slow wave (deep) sleep and reduce nocturnal awakenings (49). One meta-analysis, however, concluded that it was more likely to improve perceived sleep quality than objective measures of sleep quality (50).

*Atypical antipsychotics*, by their antagonism of histamine and serotonin receptors may also be sedating but similarly are without supportive data in the critically ill and have anticholinergic effects. Quetiapine is the third most used medication for sleep in the ICU (behind melatonin and ramelteon); however, it has mostly been studied for its effect on delirium management and has been associated with somnolence and hypotension (51). Quetiapine may increase slow wave sleep in otherwise healthy people with insomnia but these data cannot be extrapolated to the critically ill where sleep disruption is very complex and polypharmacy may lead to worse outcomes.

*Antihistamines* such as diphenhydramine may increase sedation via blockade of central H1 histamine receptors; however, they suppress REM sleep, have potent anticholinergic effects which can precipitate delirium, and their pharmacokinetics in a critically ill patient may be altered. They are not recommended for sleep promotion in the critically ill and, in fact, have mixed support for use in insomnia in non-hospitalized and non-critically ill hospitalized patients due to safety concerns including excessive sedation and persistent drowsiness likely due to their high blood-brain barrier penetration.

#### Herbal

Although not prescription medication, herbal extracts administered as aromatherapy have been tested for their effects on sleep and stress both in healthy subjects as well as those who are critically ill. One study, for example, found that in an ICU cohort, the aromatherapy group slept better than the control group although sleep quality worsened in both groups following hospitalization (52). Lavender is believed to exert its hypnotic effect by enhancing GABA signaling, increasing serotonin availability, and reducing excessive glutamate activity (53). A recent systematic review of 11 aromatherapy studies in the ICU noted low quality of evidence, however, the results suggest that a combination of lavender, Matricria recutita, and neroli essential oils improved sleep quality compared with usual care (54). In the 2018 PADIS guidelines, however, concern was raised about the use of potential respiratory irritants in an ICU environment, so a conditional recommendation was made against its use (2).

### Chronotropic realignment

It is important to acknowledge that disruption of the circadian rhythm during critical illness will not have a simple, pharmacologic fix. Its causes are multiple and its fix likely to require a multi-faceted approach. Light therapy or even just maintenance of bright light during daytime hours and darkness at night will be a necessary part of any strategy to realign patients' circadian rhythm but may not be sufficient as the sole intervention. Exercise, too, is important to circadian alignment by its effect entraining peripheral clocks and should be part of a holistic, non-pharmacologic protocol to re-establish normal circadian timing.

Another important consideration is the timing and administration of nutrition. Normally, feeding is part of what establishes the biologic rhythm in metabolically active peripheral tissue though it has little to no effect on the central clock. Chrononutrition refers to feeding directed by the biologic clock and takes into account variation in metabolism throughout the day. For example, intermittent, enteral feeding with nighttime fasting and a balanced macronutrient composition with carbohydrates, high in protein and low in fat is beneficial for circadian entrainment of the peripheral clock (55). Larger volume intermittent feeding, however, comes with an increased risk for aspiration but this approach has been insufficiently studied for its affects on outcomes.

In terms of pharmacologic approaches, *melatonin* is the primary endogenous circadian hormone. It is produced and released by the pineal gland primarily in response to ambient light. It promotes sleep via the MT1 and MT2 receptors in the suprachiasmatic nucleus and specific regions of the brain and peripheral tissues, but it is considered a chronotropic agent, not a hypnotic or sedative. It adjusts certain physiologic functions to adapt to the external environment and creates conditions that facilitate sleep including lowering core body temperature and reducing sympathetic and increasing parasympathetic tone. Its circadian effect is not dose dependent but has a mild hypnotic effect only at low doses. Further, it is an important anti-inflammatory, anti-oxidant, immune modulatory hormone.

Melatonin is often given to critically ill patients because alteration of melatonin levels is known to occur in this context, and it has a good safety profile (56). There have now been many RCT's evaluating melatonin both for its effect on sleep and to prevent delirium. A recent systematic review and meta analysis as well as the 2025 focused update of the PADIS guidelines recommends the use of melatonin for critically ill patients based on patients perceived improvement in sleep and possible reduction in the incidence of delirium although the certainty of evidence was low (30, 57). It should be noted that melatonin is not FDA-regulated and has been shown to be of varied quality and potency. A recent study found that the actual quantity of melatonin in products containing melatonin varied from 74% to 347% of the labeled quantity (58).

*Ramelteon* is a synthetic, FDA-regulated melatonin receptor agonist with higher affinity than melatonin for both the MT1 and MT2 receptors. Like melatonin, it has a favorable safety profile and minimal risk for dependence (59). In the critically ill, it has been studied more for its potential to reduce delirium then for its sleep-promoting effect. Some studies have shown a modest potential effect to reduce delirium but the quality of evidence is low (60). Results have been even more inconsistent with regard to its effect on sleep and the quality of evidence is also low (61). However, its use clinically and for research may be preferred over melatonin as a regulated, albeit more expensive, product.

The best studied of the pharmacologic options to reduce delirium include melatonin, ramelteon, and dexmedetomidine which may reduce the incidence of delirium under certain circumstances but have not consistently been shown to improve objective measures of sleep. The lack of demonstrable objective improvement in sleep may be due to the difficulty of measuring sleep during critical illness (62).

#### Wakefulness-promoting medication

Wakefulness-promoting therapies, although not specifically chronotropic agents, could be considered as an adjunct to this strategy.

Caffeine exerts its wakefulness-promoting effects by blocking adenosine, which is integral to the homeostatic drive to sleep, and increasing dopamine, norepinephrine, and acetylcholine; however, there is currently no adenosine agonist available to promote sleep.

*Modafinil* has been studied for its wakefulness promoting potential in critically ill patients (63). It is a non-amphetamine psychostimulant which enhances function in several cognitive domains and may work by increasing dopamine signaling by altering dopamine uptake and may also block reuptake of norepinephrine in the VLPO (64, 65). Further, it has been found to have anti-oxidative effects although the complete mechanism of action is unknown. It has a favorable safety profile and does not affect nocturnal sleep if given in the morning and it is not associated with rebound somnolence. A recent review of modafinil in the critically ill analyzed 10 studies involving a total of 950 patients; 3 of the studies were RCTs (63). Few major adverse effects were reported, and it has low potential for abuse, dependence, or severe drug–drug interactions although it's metabolism is dependent on the cytochrome P450 system so co-administration with some medications may affect their levels. It may be an adjunct to the care of hypoactive patients and those with a low Glasgow Coma score where excessive daytime sleepiness or low consciousness is thought to be impeding engagement and participation in cognitive and physical therapies but should undergo further study.

Other medications such as methylphenidate and amantadine have been used for disorders of consciousness in the ICU but not specifically for wakefulness promotion.

## An evidence-based approach

A holistic approach to both improving sleep and restoring circadian timing will be necessary to optimize patients' potential to get restorative sleep. Medication(s) for sleep during critical illness will not be sufficient to improve sleep or circadian disruption but can serve as an adjunct to non-pharmacologic measures.

The non-pharmacologic approach:

1. Find out the sleep habits of the patient—when they typically go to bed and wake up and if they use sleep aids. Replicate to the extent possible (66).
2. Minimize medications administered. Most medications given during critical illness have an adverse effect on sleep architecture, and polypharmacy makes drug-drug interactions more likely (67).
3. Create a sleep-friendly, calm, safe environment (2).
  a. Light needs to be on and reasonably bright during waking hours and dark during the night
  b. Noise control is very important
  c. Limit care-related interventions during the night
4. Attention to daytime schedules of activity and feeding should consider:
  a. Intermittent feeding with overnight fasting (55)
  b. Activities that include cognitive and physical activities when possible

One example of a pharmacologic approach in the ICU if the nonpharmacologic approach proves insufficient:

Melatonin, 0.5–1.0 mg or, preferably, ramelteon 8 mg 30–90 min before planned sleep time. This can be started as soon as the patient is taking oral medication. If possible, try to respect patients' normal sleep/circadian schedule

If the patient is having trouble sleeping despite the above measures and the patient IS NOT delirious, then trazadone or a DORA may be a reasonable option if the patient is able to take enteral medication.

If the patient is having trouble sleeping despite the above measures and the patient IS delirious and agitated, then consider dexmedetomidine IVI overnight. An atypical antipsychotic could be an alternative if the patient has a contraindication or adverse effect from dexmedetomidine.

For those patients whose level of consciousness, i.e., GCS, or hypoactive state limits their ability to participate in daytime activities, modafinil could be considered on a case-by-case basis.

## Sleep after the ICU

According to one recent review, sleep disturbances after ICU discharge occur in 55% of survivors at less than one month, 50% at 1–3 months, 39% at 4–6 months, 23% at 7–12 months, and 15% beyond a year (68). Some have been found to maintain their poor subjective sleep quality for at least 2 years with little improvement (69).

In addition, it is often the case that medications begun in the ICU to facilitate sleep are continued through the hospitalization and after discharge. At one large academic center, 85.6% of patients who began a new nocturnal neuroactive medication during the ICU continued to receive it on transfer to the floor and 87% of those who survived to discharge home were also continued on it (70). These medications, however, may no longer be appropriate to continue. For these patients, medication reconciliation is extremely important and is integral their post-ICU care.

After the ICU, patients should have an assessment if they are having difficulty sleeping. Evaluation for chronic insomnia includes a detailed sleep history, medication review, often a sleep diary, and sometimes testing such as actigraphy or polysomnography to investigate other possible sleep disorders.

For most, the treatment of insomnia will first include cognitive behavioral therapy for insomnia (CBT-I), the first line treatment for chronic insomnia focusing on sleep hygiene and habits favorable to good sleep. However, patients with PICS may have specific impediments to sleep, such as pain and psychosocial stress that will need to be addressed as part of a holistic strategy.

Pharmacologic therapy for these patients will need to be individualized. Melatonin or ramelteon may be a reasonable first option along with attention to sleep hygiene. For frail, elderly patients, it will still be prudent to avoid hypnotics such as the z-drugs; however, DORA's have a more favorable safety profile (71). If depression is a co-morbidity, then trazadone or doxepin may be an alternative. The choice of medication will be determined by underlying mental health challenges, side effect profile, and potential interaction with other medications the patient may be taking. The complexity of these issues may make it most appropriate to refer to a mental health professional for their expertise.

## Conclusion

Sleep is a complex process vulnerable to the physiological disruptions of critical illness and the ICU environment but essential to physical and psychological recovery. Medications used for sleep and sedation in the ICU mostly have unfavorable effects on sleep architecture but may help sleep onset and can serve as an adjunct to non-pharmacologic environmental and behavioral strategies. Following a critical illness, many patients will still struggle to restore good sleep. Individualized plans will need to be created based on their unique circumstance and challenges. In both cases, only strategies that address both circadian and homeostatic forces will be successful.
